# Limited Diagnostic Yield of Routine Gastroscopy in FIT-Positive Patients

**DOI:** 10.3390/diagnostics15212781

**Published:** 2025-11-02

**Authors:** Majd Khader, Fadi Abu Baker, Jorge-Shmuel Delgado, Avraham Yitzhak, Revital Guterman, Ruhama Elhayany, Or Bakshi, Vered Klaitman, Tali Braun, Naim Abu-Freha, Rimon Artoul

**Affiliations:** 1 Gastroenterology Institute, Assuta Medical Center, Beer-Sheva 8489507, Israel; drshmueldelgado@gmail.com (J.-S.D.); aviyi@assuta.co.il (A.Y.); revitalg@assuta.co.il (R.G.); ruhamae@assuta.co.il (R.E.); abufreha@yahoo.de (N.A.-F.); 2 Faculty of Health Sciences, Ben-Gurion University of the Negev, Beer-Sheva 8410501, Israel; bakshi_o@mac.org.il (O.B.); klaitman_v@mac.org.il (V.K.); tali_b@mac.org.il (T.B.); dr.rimon@hotmail.com (R.A.); 3 Department of Gastroenterology and Hepatology, Hillel Yaffe Medical Center, Hadera 38100, Israel; fa_fd@hotmail.com; 4 Faculty of Medicine, Technion, Haifa 3200003, Israel; 5 Maccabi Health Services South District, Beer-Sheva 8448102, Israel; 6 Geriatrics Department, Barzilai Medical Center, Ashkelon 7830604, Israel

**Keywords:** colorectal cancer, adenoma detection rate (ADR), polyp detection rate (PDR), gastroscopy, normal colonoscopy, abnormal colonoscopy, FIT

## Abstract

**Background and aim:** The necessity and diagnostic yield of routine gastroscopy in Fecal Immunochemical Test (FIT)-positive patients with normal colonoscopy findings remains controversial and poorly defined. Here, we aimed to investigate the prevalence and clinical significance of upper gastrointestinal lesions detected by gastroscopy in FIT-positive patients, stratified by normal and abnormal colonoscopy findings. **Methods:** This retrospective study included 38,392 adults (≥18 years) who tested positive for FIT between 2016 and 2022 across eight medical centers in Israel. Of them, 1560 patients underwent routine bi-directional endoscopic evaluation and were included in the final analysis. Comprehensive procedural data were retrieved, including detailed colonoscopic and gastroscopic findings. Colonoscopy outcomes included the detection of neoplastic and precancerous lesions, with the rates of adenoma and polyp detection calculated. Gastroscopy findings, including gastritis, hiatal hernia, esophagitis, duodenitis, peptic ulcer disease, and malignancy, were analyzed and compared between patients with normal and abnormal colonoscopy results. **Results:** Among 38,392 FIT-positive adults, 1560 underwent bidirectional endoscopy; of these, 632 (40.5%) had normal and 928 (59.5%) had abnormal colonoscopy findings. Gastroscopy revealed upper GI findings in both groups, with gastritis detected in 55.5% (normal colonoscopy) vs. 48.7% (abnormal colonoscopy), hiatal hernia in 15% vs. 14.9%, esophagitis in 9.0% vs. 10.3%, and duodenitis in 6.6% vs. 7.3%. Gastric ulcers were rare, observed in 0.95% of patients with normal colonoscopy and 1.29% with abnormal colonoscopy. No cases of upper gastrointestinal malignancy were detected in either group. **Conclusions:** Routine gastroscopy in FIT-positive patients demonstrates limited diagnostic yield, with clinically significant upper gastrointestinal lesions being rare.

## 1. Introduction

Colorectal cancer (CRC) is the second leading cause of cancer-related mortality worldwide and represents a growing global health burden [1,2]. Early detection and intervention are critical in reducing mortality and improving outcomes [3,4]. The fecal immunochemical test (FIT) has become a widely accepted, non-invasive screening method for CRC, offering greater sensitivity and specificity for lower gastrointestinal (GI) bleeding than traditional guaiac-based tests [5,6,7]. In Israel, FIT has been the primary CRC screening tool since approximately 2016 [8]. Following a positive FIT result, colonoscopy is the gold standard for detecting neoplastic or precancerous colorectal lesions [9,10,11,12]. However, a subset of FIT-positive patients yield normal colonoscopy results, raising critical clinical questions regarding the origin of occult bleeding [13]. In such cases, the upper GI tract may harbor significant undetected pathology without further evaluation [14,15].

Gastroscopy enables direct visualization of the esophagus, stomach, and duodenum [16,17]. Prior studies have suggested that FIT-positive patients with normal colonoscopy findings may still present with upper GI lesions, such as gastritis, ulcers, or esophagitis, which can be identified via esophagogastroduodenoscopy (EGD) [18,19]. Identifying these lesions can allow timely intervention, potentially preventing complications including persistent bleeding or progression to malignancy [20,21]. Despite this, the routine use of gastroscopy in FIT-positive individuals remains controversial due to concerns over its diagnostic yield, cost-effectiveness, and procedural burden [22,23,24,25,26].

The current study aims to evaluate the diagnostic value of gastroscopy in patients with FIT-positive results, using a large, multicenter cohort stratified by colonoscopy findings. By assessing the prevalence and clinical significance of upper gastrointestinal lesions detected through gastroscopy, we aim to inform clinical decision-making and guide future recommendations in colorectal cancer screening protocols.

## 2. Materials and Methods

### 2.1. Study Design and Data Source

The current retrospective study included 38,392 adult patients (aged 18 years or older) who tested positive for the Fecal Immunochemical Test (FIT) and subsequently underwent endoscopic evaluation between January 2016 and December 2022 at eight participating medical centers in Israel. All patient data were retrieved from institutional medical records. Detailed demographic and procedural parameters, including age, sex, and bowel preparation characteristics, were documented. Bowel preparation quality was assessed according to the Boston Bowel Preparation Scale (BBPS). A total BBPS score of 4–5 was categorized as intermediate, 6–7 as good, and 8–9 as very good. Patients with scores ≤3 were considered to have poor bowel preparation and were excluded from the analysis. Patients with incomplete demographic or procedural data were also excluded from the study. Colonoscopy findings included the detection of adenomas categorized as tubular, tubulovillous, or villous, as well as other non-adenomatous polyps. Both the polyp detection rate (PDR) and adenoma detection rate (ADR) were calculated accordingly.

ADR was defined as the proportion of colonoscopies in which at least one histologically confirmed adenomatous polyp was identified, and PDR was defined as the proportion of colonoscopies in which at least one polyp of any histological type was detected. Neoplastic lesions, such as adenocarcinoma and carcinoid tumors, and benign submucosal lesions, including leiomyomas and lipomas, were also recorded. Gastroscopy findings included diagnoses of gastritis, hiatal hernia, esophagitis, duodenitis, reflux esophagitis, peptic ulcer disease, Gastric or duodenal ulcer, severe gastritis, and malignancy. Severe gastritis was defined endoscopically as marked erythema, mucosal friability, erosions, or ulcerations extending over a large gastric area. Comparative analyses were conducted between patients with normal and abnormal colonoscopy findings to evaluate the diagnostic value of gastroscopy.

In this study, a normal colonoscopy was defined as a complete examination in which the colonoscope reached the cecum with documentation of landmarks, bowel preparation was adequate to permit reliable detection of lesions ≥ 5 mm, and the mucosa showed no pathological findings, including polyps, tumors, strictures, ulcers, or inflammatory changes. An abnormal colonoscopy was defined as any examination with one or more deviations from normal findings, such as the presence of adenomatous or serrated polyps, masses, mucosal ulceration, inflammation, diverticular disease with complications, or strictures [7,9,11].

The study protocol was reviewed and approved by the Institutional Helsinki Ethics Committee of Assuta Medical Center (Protocol No. 0049-24-ASMC, approval date: 18 August 2024).

### 2.2. Statistical Analysis

All statistical analyses were performed using GraphPad Prism version 9.0 (GraphPad Software, La Jolla, CA, USA). Continuous variables were expressed as means ± standard deviations with interquartile ranges (IQRs). Categorical variables were compared using the Chi-square test. When expected cell counts were less than 5, Fisher’s exact test was applied. Statistical significance was defined as a *p*-value < 0.05.

## 3. Results

### 3.1. FIT Patient Demographics and Population Characteristics

#### 3.1.1. Age and Gender Distribution

The median age of patients who underwent FIT was 66 years (IQR: 60.5–72.1). The gender distribution was nearly equal, with males comprising a marginally larger proportion of the patient population, at 20,078 (52.30%), compared to females, at 18,314 (47.70%).

#### 3.1.2. Bowel Preparation Quality and Effectiveness

**Figure 1:**
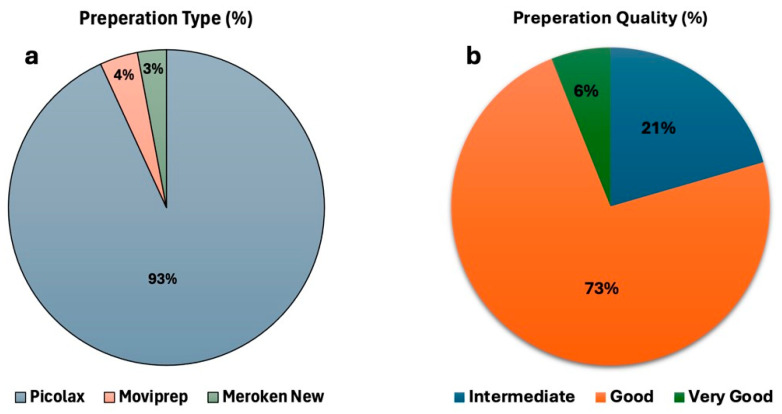
Bowel preparation type and quality. (**a**) The proportion of patients receiving different bowel preparation regimens. (**b**) The distribution of bowel preparation quality is categorized according to the Boston scale as intermediate, good, or very good.

Among the study population, Picolax (sodium picosulfate and magnesium citrate) was the most commonly used preparation regimen, with 35,766 patients (93.16%) using it. Other Polyethylene glycol (PEG)-based regimen preparations, such as Moviprep (1494 patients, 3.89%) and Meroken New (1132 patients, 2.95%), were not as widely used as shown in Figure 1a. Additionally, the overall quality of bowel preparation differed significantly among the cohort. Good or very good preparation was achieved in 79% of cases confirmed to provide the correct diagnosis. However, 21% had an intermediate level (Figure 1b).

#### 3.1.3. Adenoma Detection and Distribution

**Figure 2:**
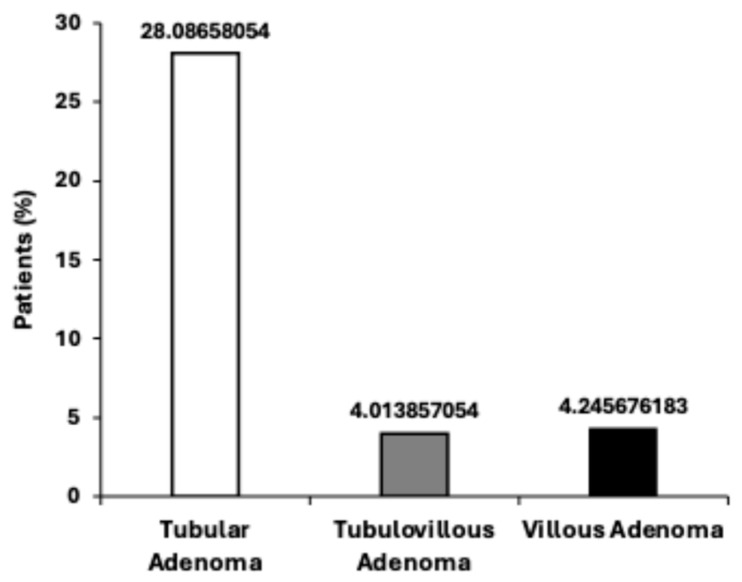
Distribution of adenoma subtypes. Bar chart illustrating the distribution of adenoma subtypes detected in the study cohort, including tubular, tubulovillous, and villous adenomas.

In the current study, 13,954 patients (36.34%) were diagnosed with adenomas, confirming the high prevalence of these precancerous lesions among individuals who were FIT-positive (Table 1). The majority of adenomas identified were tubular adenomas (10,783 patients, 28%), which are generally considered benign. However, a substantial proportion of patients presented with polyps exhibiting histological features associated with higher malignant potential, including tubulovillous adenomas (1541 patients, 4.01%) and villous adenomas (1630 patients, 4.24%) (Figure 2). As data on polyp size and dysplasia grade were unavailable, these were not classified as advanced adenomas. In the subset of 13,954 patients diagnosed with adenomas, the distribution by age showed a progressive increase with advancing age: 1395 cases (9.9%) occurred in patients younger than 50 years, 3488 cases (24.9%) in those aged 50–59 years, 4883 cases (34.9%) among patients aged 60–69 years, 2790 cases (19.9%) in the 70–79 age group, and 1398 cases (10%) among individuals aged 80 years and above (Table 2). Male patients demonstrated a significantly higher adenoma detection rate than females (*p* = 0.02), with 7674 males (38.2%) diagnosed compared to 6280 females (34.3%), as presented in Table 3.

The predominance of adenomas among older male patients increases the need for focused screening in these high-risk groups. In addition, the prevalence of tubular adenomas justifies the surveillance of patients found positive for FIT, whereas the existence of villous and tubulovillous adenomas in a small, yet clinically significant subset, confirms the necessity of close monitoring and timely intervention for high-risk lesions to prevent their malignant transformation.

#### 3.1.4. Polyp Detection Rate (PDR) and Distribution

**Figure 3:**
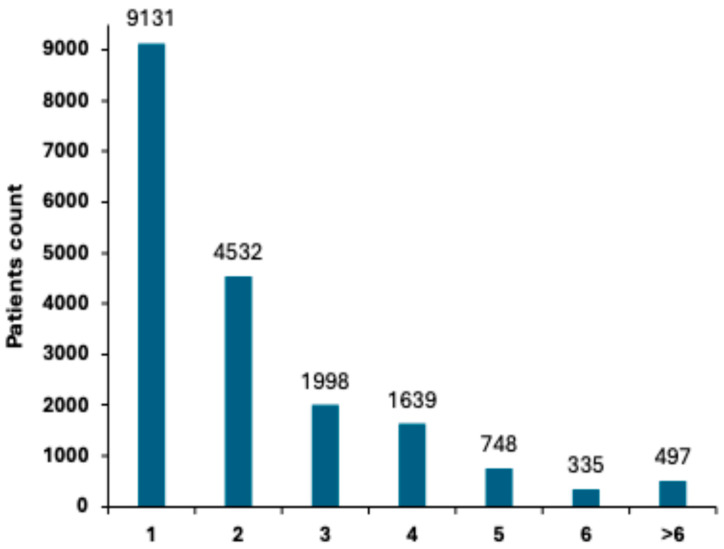
Distribution of polyp count per patient. Bar chart showing the number of polyps detected per patient in the study cohort.

In our cohort, 18,880 patients were diagnosed with at least one polyp, resulting in a Polyp diagnostic rate (PDR) of 49.1% (Table 4), indicating that nearly half of the screened patients had polyps. Additional analysis, based on the stratification of the number of polyps detected per patient, presented in Figure 3, demonstrated that 9131 patients (23.8%) had one polyp and 4532 (11.8%) had two polyps. As the number of polyps increased, the prevalence decreased, with 1998 (5.2%) having three polyps, 1639 (4.26%) having four polyps, and 748 (1.94%) having five polyps. A small group of 832 patients (4.4%) had six or more polyps, representing an increased risk group that requires closer monitoring (Figure 3).

#### 3.1.5. Colorectal Neoplasms and Tumor Distribution

**Figure 4:**
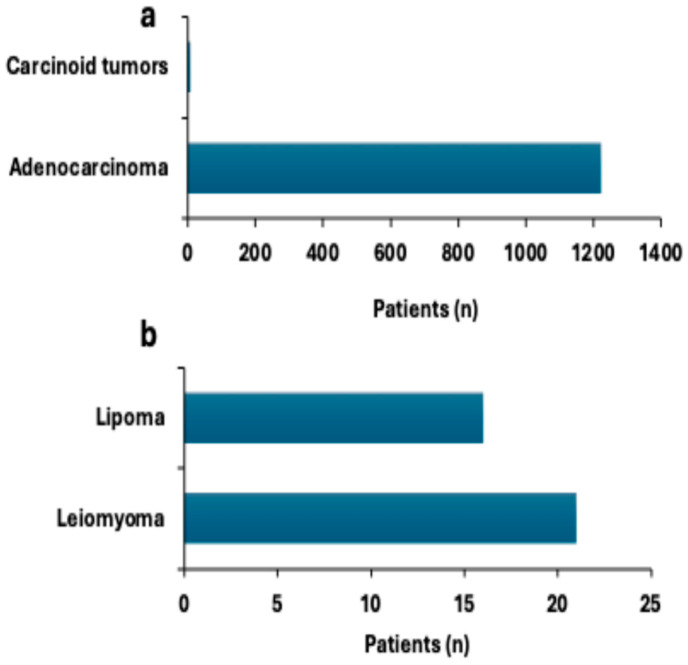
Prevalence of colorectal tumors and submucosal lesions. (**a**) The distribution of malignant colorectal tumors, including adenocarcinoma and carcinoid tumors, is presented as a percentage of the study population. (**b**) The prevalence of submucosal lesions, including lipomas and leiomyomas, is presented as a percentage of the total patient population.

Of the malignant lesions, adenocarcinoma was the most frequently found type of cancer, which was documented in 1222 patients (Figure 4a). In six patients, carcinoid tumors, which are a rare form of neuroendocrine malignancy, were noted, demonstrating their low frequency in colorectal pathology (Figure 4a). In the case of benign neoplasms, leiomyomas were documented in 21 patients and lipomas in 16 patients (Figure 4b). Although these lesions are not malignant and do not usually present a high risk for cancer, the indolent ones can be clinically significant, especially when symptomatic or complicated. Adenocarcinoma was the most common colorectal malignancy identified in this cohort, underscoring the need for early detection and treatment. Carcinoid tumors and benign lesions are less common; these tumors still require recognition in the context of comprehensive patient care.

#### 3.1.6. Prevalence of Gastrointestinal Disorders

**Figure 5:**
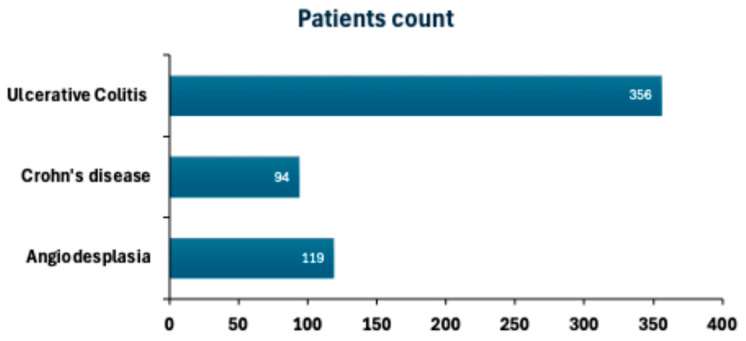
Prevalence of colonic vascular and inflammatory conditions. Bar chart illustrating the percentage of patients diagnosed with Ulcerative colitis, Crohn’s disease, and angiodysplasia within the study cohort.

Within the studied patients, Ulcerative colitis was the most commonly diagnosed condition, affecting 356 patients (0.93%, Figure 5). Angiodysplasia, a recognized cause of gastrointestinal bleeding, was identified in 119 patients (0.31%), with Crohn’s disease being the least prevalent, affecting 94 patients (0.24%), as shown in Figure 5. These results indicate that Ulcerative colitis was the most common gastrointestinal disorder (0.93%), while Crohn’s disease and angiodysplasia were less frequent.

#### 3.1.7. Gastric Outcomes in FIT-Positive Patients Undergoing Both Colonoscopy and Gastroscopy

**Figure 6:**
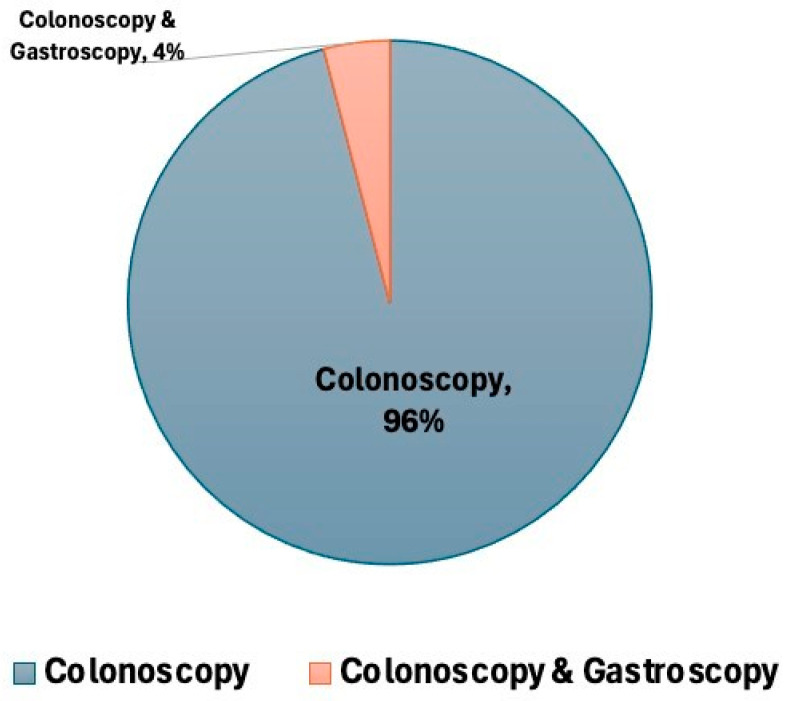
Proportion of patients undergoing colonoscopy alone versus combined colonoscopy and gastroscopy. Pie chart depicting the distribution of patients who underwent colonoscopy alone compared to those who underwent both colonoscopy and gastroscopy.

**Figure 7:**
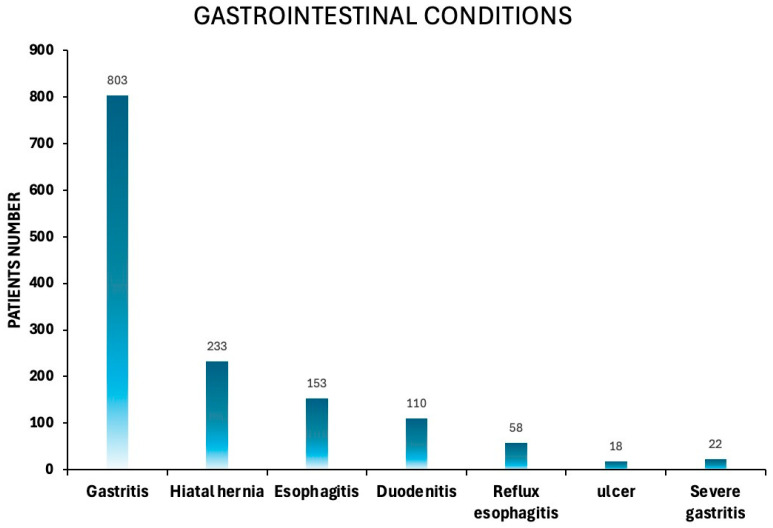
Distribution of upper gastrointestinal findings among patients undergoing gastroscopy. Bar chart illustrating the prevalence of various upper gastrointestinal conditions, including gastritis, hiatal hernia, esophagitis, duodenitis, reflux esophagitis, ulcers, and severe gastritis.

Among the total cohort of patients with a positive FIT, 1560 individuals (4.06% of the total population) underwent further evaluation (Figure 6). Of these, 773 (49.55%) were male and 787 (50.44%) were female. The cohort’s mean age was 66.28 ± 8.59, and the median age was 66 years. A considerable number of patients in this study were found to have upper gastrointestinal disorders (Figure 7), reflecting the underlying prevalence of such conditions in the screened population. Gastritis was the most frequently observed condition, affecting 803 patients (51.47%). Hiatal hernia was found in 233 patients (14.9%). Moreover, esophagitis was reported in 153 patients (9.81%). Additionally, 110 patients (7.05%) were diagnosed with duodenitis, and 58 patients (3.72%) with reflux esophagitis. Fewer patients had more severe lesions, as 22 (1.41%) patients had severe gastritis and 18 (1.15%) patients had peptic ulcer disease (Figure 7).

#### 3.1.8. Gastroscopy as a Key Diagnostic Tool in FIT-Positive Patients with Normal Colonoscopy Findings

**Figure 8:**
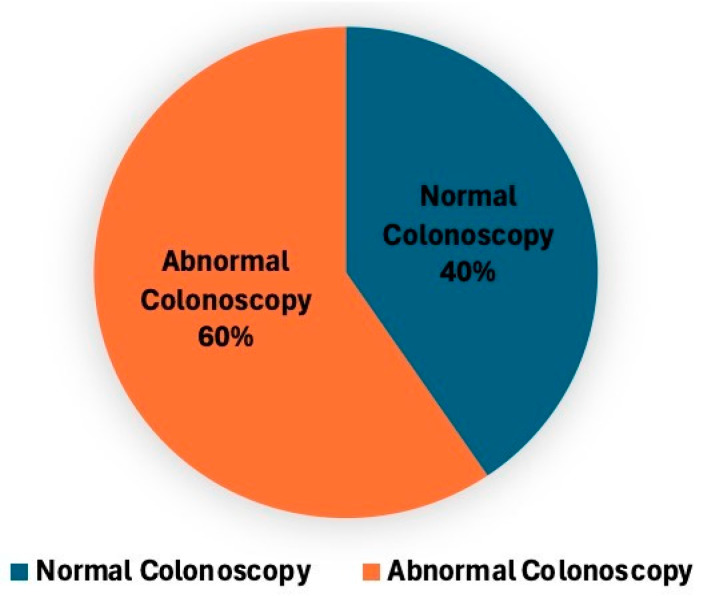
Proportion of normal and abnormal colonoscopy findings among patients undergoing colonoscopy and gastroscopy. Pie chart depicting the distribution of colonoscopy results, showing the proportion of patients with normal versus abnormal findings.

**Figure 9:**
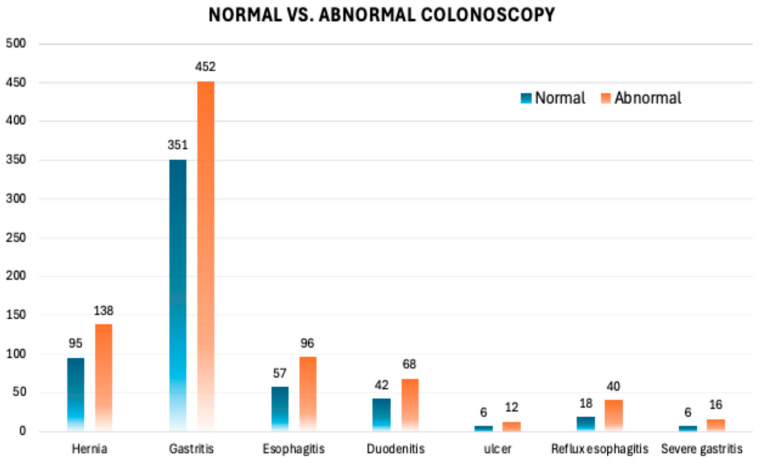
Comparison of normal and abnormal gastroscopy findings. Bar chart illustrating the distribution of various upper gastrointestinal conditions, categorized as normal or abnormal findings. Conditions include hernia, gastritis, esophagitis, duodenitis, ulcers, reflux esophagitis, and severe gastritis.

To further investigate the clinical value of performing gastroscopy in FIT-positive patients, we stratified the population based on colonoscopy findings into two groups: normal colonoscopy (*n* = 632, 40.5%) and abnormal colonoscopy (*n* = 928, 59.5%) (Figure 8). The primary objective of this analysis is to determine whether upper GI pathology differs between these two subgroups and whether gastroscopy is particularly valuable in patients with a normal colonoscopy, particularly in those with FIT-positive results. Among the most prevalent findings shown in Figure 9, gastritis was diagnosed in 351 patients (55.5%) in the normal colonoscopy group and 452 patients (48.7%) in the abnormal colonoscopy group (*p* = 0.04). Hiatal hernia was identified in 95 (15%) patients in the normal colonoscopy group, compared to 138 (14.9%) patients in the abnormal colonoscopy group, showing a comparable distribution between the two groups (*p* = ns). Similarly, esophagitis and reflux esophagitis were similar in both subgroups, affecting 96 patients versus 57 and 40 patients versus 18, respectively (*p* = ns). Additionally, duodenitis was detected in 42 (6.6%) vs. 68 (7.3%) patients. Finally, significant findings were rarely observed in ulcers (0.95% vs. 1.29%), severe gastritis, and were entirely absent in cases of gastric cancer. Although gastritis was statistically more common in the normal colonoscopy group (*p* = 0.04), this difference is not clinically relevant, as gastritis was prevalent in both groups and rarely associated with severe pathology. All statistical calculations are presented in Table A1. Our data showed that gastroscopy demonstrated a comparable prevalence of upper GI pathology in FIT-positive patients, regardless of colonoscopy findings.

## 4. Discussion

Several studies have investigated the diagnostic value of performing gastroscopy in FIT-positive patients who have already undergone colonoscopy, yielding findings that present both supportive and opposing views. Several studies have shown that adding upper endoscopy can identify upper-GI lesions in a meaningful proportion of FIT-positive individuals, supporting a selective role for gastroscopy in this setting [12,13,18]. Similarly, Planade et al. demonstrated that nearly 48% of FIT-positive individuals had clinically relevant upper GI findings uncovered during concurrent gastroscopy, influencing subsequent management [18]. A meta-analysis by Shah et al. further reinforced this observation, concluding that bi-directional endoscopy significantly improves diagnostic yield, particularly for conditions such as gastritis, ulcers, and duodenitis [13]. Other supportive evidence comes from Kay, C. L. et al., who emphasized the added value of same-day upper endoscopy in cases of unexplained positive FIT results [27]. Burri et al. also highlighted the diagnostic enhancement provided by gastroscopy in patients with vague abdominal complaints [21]. At the same time, Lee et al. confirmed that FIT may detect bleeding from upper GI sources, not just colorectal origins [15].

In contrast, other researchers argue against the routine use of gastroscopy in populations with FIT-positive results. Ng et al. found that clinically significant upper GI lesions were rare in this cohort, which raises questions about the utility and cost-effectiveness of widespread use of gastroscopy [28]. Likewise, McLoughlin & Telford and Allard et al. observed that gastroscopy seldom influenced patient management when colonoscopy results were normal, recommending a more selective approach based on clinical presentation [24,25]. Doubeni et al. emphasized that FIT is primarily sensitive to lower GI bleeding, and upper endoscopy should be reserved for patients with upper GI symptoms or known risk factors [6]. Elsafi et al. echoed this sentiment, suggesting that most FIT false positives are not attributable to upper GI lesions, making routine gastroscopy in asymptomatic individuals potentially low-yield [14]. These contrasting perspectives highlight the ongoing debate in clinical practice and underscore the need for individualized, risk-based decision-making when evaluating patients with FIT-positive results and normal colonoscopy findings.

Our results align more closely with the latter group of studies, suggesting that routine gastroscopy in FIT-positive patients with normal colonoscopy findings has limited diagnostic value, as the prevalence of clinically severe or significant upper gastrointestinal pathology was minimal, with rare ulcers and no cases of gastric cancer identified. Therefore, routine gastroscopy does not appear warranted for all FIT-positive patients. Instead, decisions regarding further upper gastrointestinal evaluation may be better guided by clinical judgment on a case-by-case basis, considering individual patient symptoms and risk factors. This approach may prevent unnecessary procedures, minimize patient burden, and optimize the utilization of healthcare resources. A limitation of this study is that detailed histopathologic data were not uniformly available in the retrospective records. As a result, abnormal colonoscopic findings could not be further classified into non-invasive and invasive categories, which may have limited the granularity of lesion characterization. Another limitation of this study is that multivariate analysis could not be performed to adjust for potential confounders such as comorbidities, medication use, or other clinical variables, as these data were not uniformly available in the retrospective records.

Furthermore, our study’s strengths include a large and diverse cohort from eight medical centers, robust statistical comparisons, and real-world relevance. However, it is essential to acknowledge its limitations, including its retrospective design and the lack of data on symptoms, medication use (e.g., NSAIDs or PPIs), and H. pylori status, which may have influenced the gastroscopy findings.

Our findings support a more selective approach to upper gastrointestinal evaluation following a positive FIT. Given the very low rate of clinically significant upper GI pathology observed, routine gastroscopy in all FIT-positive individuals with normal colonoscopy findings does not appear justified. Instead, screening guidelines could consider recommending gastroscopy only for subgroups with specific risk factors, such as age above 60 years, chronic PPI use, anemia, or persistent upper GI symptoms. Incorporating these stratified criteria into national CRC screening protocols could improve cost-effectiveness while minimizing unnecessary procedures

## Figures and Tables

**Table 1:** Adenoma detection rate of the whole cohort.

Patients with Adenoma (n)	ADR (%)
13,954	36.34

**Table 2:** Numbers and percentages of cases with adenoma by age.

Age Group (Years)	Number of Cases with Adenoma	(%)
<50	1395	9.9
50–59	3488	24.9
60–69	4883	34.9
70–79	2790	19.9
≥80	1398	10

**Table 3:** Adenoma Detection Rate by Gender.

Gender	Number of Cases with Adenoma	ADR (%)
Male (*n* = 20,078)	7674	38.2
Female (*n* = 18,314)	6280	34.3

**Table 4:** Polyps detection rate of the whole cohort.

Patients with Polyps (n)	PDR (%)
18,880	49.1

## Data Availability

The datasets used and/or analyzed during the current study are available from the corresponding author on reasonable request.

## Appendix A

**Table A1:** Statistical comparison (*p*-values) of upper gastrointestinal findings in FIT-positive patients with normal versus abnormal colonoscopy results (corresponding to Figure 9). This table summarizes the *p*-values derived from Chi-square or Fisher’s exact tests comparing the frequency of each gastroscopic finding (e.g., gastritis, hernia, esophagitis, duodenitis, ulcers, reflux esophagitis, and severe gastritis) between the two groups, * *p* < 0.05 indicates statistical significance.

Gastric Finding	*p*-Value
Hernia	0.92377
Gastritis	0.040289 *
Esophagitis	0.327184
Duodenitis	0.559483
Ulcer	0.654623
Reflux esophagitis	0.138902
Severe gastritis	0.260343
